# Evaluating the optimal re-irradiation dose for locally recurrent esophageal squamous cell carcinoma after definitive radiotherapy

**DOI:** 10.1186/s13014-019-1402-1

**Published:** 2019-11-04

**Authors:** Xiujuan Xu, Zhongming Wang, Shunian Jiang, Yuping Shang, Yan Wu

**Affiliations:** Department of Radiation Oncology, Lianyungang No 2 People’s Hospital, Lianyungang, 222023 China

**Keywords:** Esophageal squamous cell carcinoma, Local recurrence, Prognostic factors, Re-irradiation dose

## Abstract

**Background:**

Re-irradiation (re-RT) has the active effect of relieving clinical symptoms and prolonging the survival of patients with recurrent esophageal squamous cell carcinoma (ESCC). However, the optimal re-RT dose is still uncertain. Here, we analyzed the prognostic factors associated with survival and explored the optimal re-RT dose for patients with recurrent ESCC following definitive radiotherapy.

**Patients and methods:**

The data of 47 patients with recurrent ESCC who were retreated between 2010 and 2014 were retrospectively analyzed. All patients received a radiation dose > 50 Gy during the primary treatment. The median time to recurrence after primary radiotherapy was 26 months (range 6–120 months). All patients had in-field recurrence in the esophagus. Recurrence within the local site was observed in 37 patients (78.7%), and recurrence in both the local site and regional nodes were observed in 10 patients (21.3%). All patients received 3D conformal re-RT with a median dose of 58 Gy (range 26–64 Gy). Chemotherapy was sequentially used in 27.7% of the patients. Survival curves were constructed according to the Kaplan-Meier method and were compared by log-rank tests. The factors predictive of survival were identified with univariate and multivariate analyses.

**Results:**

Dysphagia relief after re-RT was achieved in 20 of the 35 symptomatic patients (57.1%). The median survival time (MST) of all patients was 17 months, and the 1-, 2-, 3- and 5-year overall survival (OS) rates were 72.3, 25.5, 17.0 and 2.1%, respectively. In the univariate analysis, an Eastern Cooperative Oncology Group Performance Status (ECOG-PS) of 0–1 (*P* = 0.014), recurrence at the local site (*P* = 0.048), time to recurrence ≥24 months (*P* = 0.006) and re-RT dose ≥50 Gy (*P* < 0.001) were associated with favorable OS. In the multivariate analysis, only re-RT dose was an independent factor for OS (*P* = 0.007). Severe complications were observed in 7 patients, two of whom received a re-RT dose > 60 Gy.

**Conclusion:**

Our results demonstrated that patients with recurrent ESCC following definitive radiotherapy had unfavorable OS. Re-RT could be considered a feasible and effective treatment modality. A re-RT dose > 50 Gy could improve the survival outcomes, and a dose > 60 Gy should be administered with caution due to the risk of severe complications.

## Background

Esophageal squamous cell carcinoma (ESCC) is a common type of malignant tumor in the Northern Jiangsu Province, and chemoradiotherapy is a viable definitive treatment for inoperable ESCC [1]. Local recurrence is a major problem for the failure of ESCC after definitive chemoradiotherapy, and occurs in approximately 40–60% of patients [2, 3]. The treatments for recurrent ESCC include salvage surgery, endoscopic treatment, radiotherapy, chemotherapy or combined methods. Salvage surgery is considered the most effective treatment, but only carefully selected patients can undergo surgery due to the high operative mortality rate and risk of pulmonary complications [4, 5]. Endoscopic therapy is an effective and less invasive option for locally recurrent esophageal carcinoma after definitive radiotherapy, but careful patient selection is crucial [6]. Chemotherapy only is usually performed for patients with metastatic disease. Therefore, re-irradiation (re-RT) remains a common salvage treatment for recurrent ESCC after primary radical radiotherapy or chemoradiotherapy. It has been shown that re-RT has an active effect on relieving clinical symptoms and prolonging survival, similar to salvage surgery [7]. However, the optimal re-RT dose for local recurrent ESCC is still uncertain. In our study, we retrospectively analyzed the prognostic factors associated with survival and explored the optimal re-RT dose for patients with locally recurrent ESCC following definitive radiotherapy or chemoradiotherapy.

## Patients and methods

### Patient population

Between January 2010 and December 2014, 47 patients with recurrent ESCC following definitive radiotherapy who were retreated with re-RT were studied retrospectively. All patients received a radiation dose > 50 Gy in the initial treatment with conventional two-dimensional radiotherapy. The patients all had histologically proven in-field recurrence, with no distant metastases. Recurrence within the local site was observed in 37 patients (78.7%), and recurrence in both the local site and regional lymph nodes was observed in 10 patients (21.3%). Thirty-five patients were male, and 12 were female. The ages of the patients ranged from 54 to 83 years, with a median of 72 years. At the time of diagnosis, 35 patients (74.5%) had dysphagia, 6 patients had chest pain, 2 patients had hoarseness, and the other 4 patients had no symptoms. The median time to recurrence after primary radiotherapy for all patients was 26 months (6–120 months). Patients who received surgical therapy or brachytherapy were excluded. Patient characteristics are shown in Table 1.

**Table 1:** Characteristics of all patients

Characteristics	All patients
Age, median (range) years	72(54~83)
Sex, n (%)	
Male	35(74.5)
Female	12(25.5)
ECOG-PS	
0	8(17.0)
1	16(34.0)
2	20(42.6)
3	3(6.4)
Initial site, n (%)	
Cervical	7(14.9)
Upper thoracic	13(27.7)
Middle thoracic	18(38.3)
Lower thoracic	9(19.1)
Recurrence pattern, n (%)	
Local	37(78.7)
Local + nodal	10(21.3)
Time to recurrence median (range) months	26(6–120)
Chemotherapy, n (%)	
Yes	13(27.7)
No	34(72.3)
Re-RT dose median, (range) Gy	58(26~64)

*ECOG-PS* Eastern Cooperative Oncology Group Performance Status, *Re-RT* re-irradiation

### Treatment and follow-up

All patients received 3D conformal radiotherapy with external beam radiation using 6–15 MV linear accelerators, 1.8–2.0 Gy per fraction, 5 days/week. The planning target volume (PTV) was defined as the site of disease with a 1–2 cm margin, and the lymphatic drainage was not included in the treatment field. The re-RT doses ranged from 26 to 64 Gy, with a median of 58 Gy. Doses < 50 Gy were administered to 15 patients, 50–60 Gy to 29 patients and > 60 Gy to 3 patients. There were 9 patients who had interrupted re-RT and received doses ≤40 Gy due to major bleeding (1 patient), esophageal fistula (1 patient), rapid progression (3 patient) and cost of treatment concerns (4 patient). Only 13 patients (27.7%) received 2–4 cycles of chemotherapy (cisplatin-based chemotherapy regimen) sequentially after re-RT.

Toxicity was assessed according to the RTOG/ EORTC scoring criteria and the Common Terminology Criteria for Adverse Events version 3.0 (CTCAE v3.0). Follow-up evaluations with CT, barium meal and/or endoscopy were performed every 3 months for the first 2 years and every 6 months thereafter.

### Statistical methods

The time to recurrence was calculated from the end date of the initial radiotherapy session to the date of recurrence. Overall survival (OS) was calculated from the start da date ta of re-RT to the date of death or last follow-up. Survival curves were constructed according to the Kaplan-Meier method, and differences in the curves were compared by log-rank tests. A multivariate analysis of the prognostic factors was performed using Cox regression models. *P*-values less than 0.05 were considered statistically significant. Analyses were performed using SPSS ver. 18.0 (SPSS Inc., Chicago, IL).

## Results

### Treatment outcomes

**Fig. 1:**
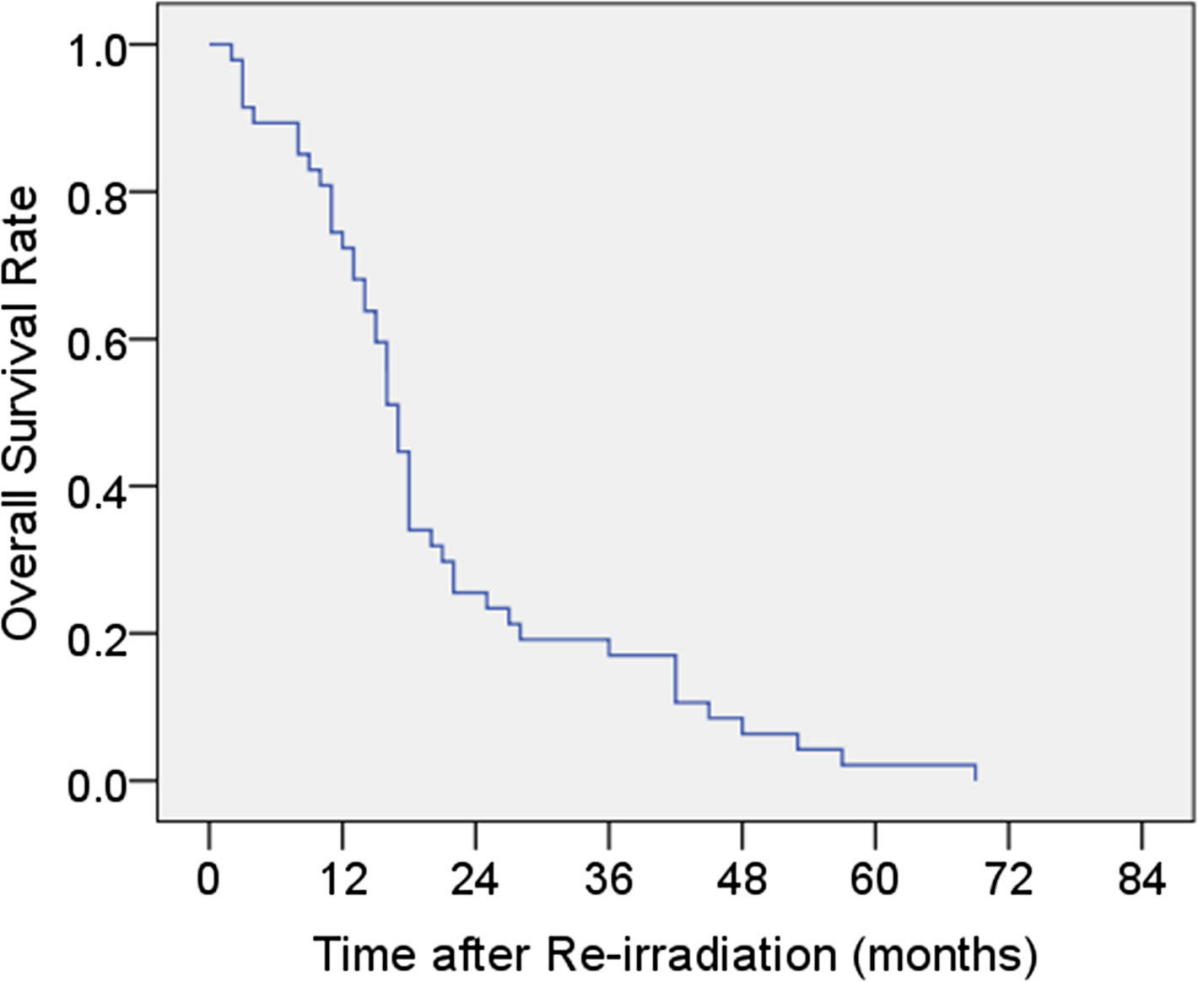
Kaplan-Meier survival analysis of all patients

The last follow-up was July 2019. The follow-up periods ranged from 1 to 68 months (median: 16 months). At the time of analysis, all the patients had died. At the time of 6–8 weeks after re-RT, dysphagia relief was achieved in 20 of the 35 symptomatic patients (57.1%). The 1-, 2-, 3- and 5-year OS rates were 72.3, 25.5, 17.0 and 2.1%, respectively, with a median survival time (MST) of 17 months (95%CI: 15.1–18.9). Figure 1 shows the OS curve after re-RT for all patients.

### Clinical prognostic factors for survival

**Fig. 2:**
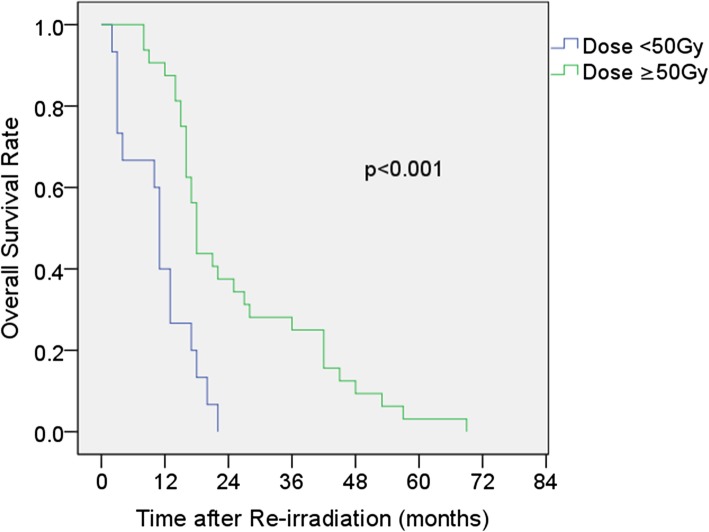
Kaplan-Meier survival analysis comparing re-RT doses < 50 Gy and ≥ 50 Gy

In the univariate analysis, an ECOG-PS of 0–1 (*P* = 0.014), recurrence at the local site (*P* = 0.048), time to recurrence ≥24 months (*P* = 0.006) and re-RT dose ≥50 Gy (*P* < 0.001) were associated with favorable OS (Table 2). We examined the possible prognostic factors (age, sex, ECOG-PS, recurrence pattern, time to recurrence and re-RT dose), and a multivariate analysis was performed. Only a re-RT dose ≥50 Gy was an independent prognostic factor for OS (*P* = 0.006). The MST and the 2-year OS rate for patients treated with dosed< 50 Gy were 11 months (95%CI: 9.8–12.2) and 0% compared with 18 months (95%CI: 16.6–19.4) and 37.5% (95%CI: 20.6–54.4%) for those treated with doses ≥50 Gy (*P* < 0.001) (Fig. 2).

**Table 2:** Univariate analyses of the prognostic factors for survival

Factor	No.	MST (months)	2-year OS(%)	95%CI	*P* value
Age					
< 70	18	16	33.3	11.5–55.1	0.070
≥70	29	17	20.7	6.0–35.4	
Sex					
Male	35	16	20.0	6.7–33.3	0.083
Female	12	17	41.7	13.9–69.5	
ECOG-PS					
0–1	24	18	37.5	18.1–56.9	0.014
2–3	23	15	13.0	0.26–25.74	
Initial site					
Cervical +upper thoracic	20	17	30.0	10.0–50.0	0.100
Middle thoracic	18	16	33.3	11.5–55.1	
Lower thoracic	9	12	0	–	
Recurrence pattern					
Local	37	17	32.4	17.3–47.5	0.048
Local +nodal	10	14	0	–	
Time to recurrence					
< 24 months	19	12	15.8	0.5–31.1	0.006
≥24 months	28	18	32.1	14.9–49.3	
Chemotherapy					
Yes	13	18	23.1	0.2–46.0	0.812
No	34	16	26.5	11.6–41.4	
Re-RT dose					
<50Gy	15	11	0	–	< 0.001
≥50Gy	32	18	37.5	20.6–54.4	

*ECOG-PS* Eastern Cooperative Oncology Group Performance Status, *Re-RT* re-irradiation, *MST* median survival time, *OS* overall survival, *CI* confidence interval

### Toxicity

Radiation esophagitis and leukocytopenia are common acute toxicities and can be manageable. Radiation esophagitis occurred in 34 patients (grade 1–2 = 27 patients, grade 3–4 = 7 patients), and leukocytopenia occurred in 26 patients (grade 1–2 = 19 patients, grade 3 = 7 patients). Grade 1–2 radiation pneumonitis and stenosis of the esophagus were noted in 4 and 11 patients, respectively. No radiation myelitis or cardiac injuries were observed in the study.

Severe complications were observed in 7 patients. One patient died of major bleeding 1 month after the end of re-RT with a dose of 34 Gy. Four patients developed esophageal fistulas. One patient developed an esophageal fistula 5 months after the completion of re-RT with a dose of 60 Gy and underwent gastrostomy; two patients developed esophageal fistulas during re-RT with doses of 34 Gy and 46 Gy, and both of these patients terminated gastrostomy, esophageal stent implantation and further anti-tumor therapy. The fourth patient developed an esophageal fistula 2 months after the completion of re-RT with a dose of 64 Gy and underwent gastrostomy. Grade 3 stenosis of the esophagus was observed in 2 patients who received doses of 60 Gy and 64 Gy. Both patients finally died of hypoproteinemia and a water-electrolyte imbalance.

## Discussion

Local recurrence after definitive radiotherapy or chemoradiotherapy is a major problem for patients with ESCC; the prognosis of these patients is very poor, and most of these patients will die in 1 year without treatment [7–9]. Re-RT plays a prominent role in the treatment of locally recurrent ESCC and has been reported to have beneficial effects on symptomatic control and curative potential [7, 10, 11]. Chen analyzed the treatment modalities for recurrent ESCC following definitive radiotherapy and compared the survival of patients who received salvage surgery, chemoradiation and best supportive care. All of the patients who received best supportive care died within 1 year. However, the 1-, 3-, and 5-year survival rates for patients who underwent salvage chemoradiation were 51.7, 12.2 and 3.1%, respectively, which are comparable to those of patients who underwent salvage surgery [7]. Fakhrian reported that 2 patients who received initial treatment with radiotherapy lived for more than 5 years after being treated with re-RT. Relief of the symptoms was achieved in 19 of the 28 symptomatic patients (68%) [11]. Katano described 6 patients who underwent re-irradiation for locally recurrent esophageal cancer following definitive chemoradiotherapy. The MST after re-irradiation was 13.6 months (range, 1.9–33.3 months) [10]. Hong also reported that re-irradiation could improve the long-term survival of patients with locoregional recurrent ESCC, with a MST of 21 months and a 5-year OS of 13.08% [12]. This outcome was better than that in other studies, and it should be considered that in Hong’s study, 16.1% of the patients had regional lymph node recurrence only.

In our study, the majority of the patients (74.5%) had dysphagia at the time of diagnosis, and approximately 57.1% had palliation of dysphagia after re-RT. These data are comparable with studies on patients with ESCC undergoing definitive chemoradiotherapy [8]. However, the prognosis of these patients is poor, with only one patient living for more than 5 years and most patients dying within 2 years. The 1-, 2-, 3- and 5-year OS rates were 72.3, 25.5, 17.0 and 2.1%, respectively, with a MST of 17 months. These data are comparable with a multi-institutional study from Japan [13] that reported the effectiveness and toxicity of re-RT for patients with oligo-recurrence in lymph nodes from esophageal cancer and compared the outcomes of patients who did and did not receive previous radiation; the study found that compared to patients without an irradiation history, patients with a past irradiation history have a poorer prognosis, with a MST and 3-year OS of 16.0 months and 17.9%, respectively. In our study of re-treatment, although most of the toxicities were manageable, seven patients still developed severe complications, including one patient who died of major bleeding, two who had grade 3 stenosis of the esophagus, and four who had esophageal fistula.

A higher re-RT dose has been recommended for recurrent lung cancer, nasopharyngeal carcinoma and other tumors and has been shown to lead to an increase in local control and survival [14–16]. However, the optimal re-RT dose in patients with recurrent ESCC following definitive radiotherapy remains uncertain, although the importance of re-RT is well known. In a retrospective study, Fakhrian reported that 54 patients with recurrent esophageal carcinoma underwent salvage radiotherapy with a median dose of 45 Gy (range 30–68 Gy). These results suggest that a minimum total dose of 45 Gy is recommended. No treatment-related deaths were observed [11]. Kim analyzed 10 patients with recurrent esophageal cancer treated with a re-RT dose of 44–50.4 Gy after primary definitive radiotherapy, and two patients had complete response [17]. Chemoradiotherapy was performed in Chen’s study, and intensity-modulated radiation therapy (IMRT) for recurrent ESCC was administered in a total dose of 50.4 Gy. The outcomes were similar to those of salvage surgery, but the frequency of complications was higher in patients who received IMRT [7]. Zhou also noted that a salvage radiation dose > 50 Gy yielded better survival for patients with recurrent esophageal cancer, and the toxicities were tolerable [18]. In our study, the re-RT dose was an important prognostic factor in univariate and multivariate analyses. The MST of patients who received a dose ≥50 Gy was 18 months (95%CI: 16.6–19.4) and that of patients who received < 50 Gy was 11 months (95%CI: 9.8–12.2); this result is similar to those in other reports.

ESCC is common in Asian populations, and a dose > 60 Gy in the initial treatment of inoperable esophageal cancer yielded a better improvement in survival [19]. Most studies have shown that a radiation dose > 50 Gy yielded better survival for recurrent ESCC, but the studies did not mention the survival rates and toxicity at the relatively higher dose. Due to the need to protect normal tissues, individual differences, patterns of recurrence and variations in initial treatment, the optimal re-RT dose is difficult to decide for recurrent ESCC. Would a dose > 60 Gy be favorable? In our study, 3 patients received a re-RT dose > 60 Gy, two of whom developed severe complications. One patient developed an esophageal fistula and finally died of a chest infection. The other patient developed grade 3 stenosis of the esophagus and died of hypoproteinemia and a water-electrolyte imbalance. It seems that patients who received a dose > 60 Gy had a high incidence of severe complications. Chen et al. [7] reported that esophageal fistulas were more frequent in patients who received a dose of 63 Gy than in patients who received a dose of 54 Gy; the authors also found that in patients who underwent salvage surgery, anastomotic leakage occurred more frequently in patients who received a dose of 63 Gy than in patients who received a dose of 54 Gy. A re-RT dose lower than the primary radiation dose was also suggested for locally recurrent nasopharyngeal carcinoma, and a higher re-RT dose could result in more complications [20]. Thus, a re-RT dose > 60 Gy should be administered with caution, as severe complications may seriously affect the quality of life and prognosis of patients with recurrent ESCC following definitive radiotherapy.

Similar to the study from Zhou [18], our study reported that the survival rate increased with longer times to recurrence, and patients with a time to recurrence ≥24 months had a better OS. Kim described 10 patients who underwent re-irradiation for locally recurrent esophageal cancer following definitive radiotherapy, and the 2 patients who had long times to recurrence (17.8 and 36.4 months) had a complete response [17]. This finding has also been reported in many studies of other tumors [14, 16, 21]. However, Hong failed to show the association between survival and time to recurrence [12], probably because the study included patients with mixed patterns of recurrence, including those with regional lymph node recurrence only, local failure or both.

The poor PS of patients is one of the causes of poor prognosis, and it has been reported that locally advanced ESCC patients with an ECOG-PS of 0–1 had better OS than patients with an ECOG-PS of 2 in the initial treatment of chemoradiotherapy [8]. In our study, we also obtained a similar result: the 2-year OS rate of patients with an ECOG-PS of 0–1 was 37.5% (95%CI: 18.1–56.9%) and that of patients with an ECOG-PS of 2–3 was 13.0% (95%CI: 0.26–25.74%) (*P* = 0.014). Some studies reported that local recurrence within the primary site without regional lymph node involvement was associated with favorable OS [11, 12]. In our study, the recurrence pattern could also be a prognostic factor for OS (local vs. local +nodal, MST 17 months vs. 14 months, *P* = 0.048). Usually, chemoradiotherapy is recommended for ESCC as initial treatment. Due to the patient age in our study (median 72 years), only 27.7% of our patients received 2–4 courses of chemotherapy, and the advantage of chemotherapy was not significant for survival.

In our study, all patients received 3D conformal radiotherapy, and further investigations of IMRT are needed to better analyze the prognostic factors and explore the optimal re-RT dose for patients with local recurrent ESCC. Due to the small sample size and retrospective nature of this study, it is necessary to further analyze a larger series of patients.

## Conclusion

In conclusion, the prognosis of recurrent ESCC is poor. Re-RT is an important approach to improving the outcomes for locally recurrent ESCC after definitive radiotherapy. A re-RT dose > 50 will be helpful in prolonging survival, and a dose > 60 Gy should be carefully administered due to the risk of severe complications.

## Data Availability

The datasets used and/or analyzed during the current study are available from the corresponding author on reasonable request.

## Abbreviations

CI: Confidence interval
CT: Computed tomography
CTCAE v3.0: Common Terminology Criteria for Adverse Events version 3.0
ECOG-PS: Eastern Cooperative Oncology Group Performance Status
ESCC: Esophageal squamous cell carcinoma
IMRT: Intensity-modulated radiation therapy
MST: Median survival time
OS: Overall survival
PTV: Planning target volume
Re-RT: Re-irradiation
RTOG/ EORTC: The Radiation Therapy Oncology Group/ European Organization for Research on Treatment of Cancer
